# Comprehensive Analysis of lncRNAs and circRNAs Reveals the Metabolic Specialization in Oxidative and Glycolytic Skeletal Muscles

**DOI:** 10.3390/ijms20122855

**Published:** 2019-06-12

**Authors:** Linyuan Shen, Mailin Gan, Qianzi Tang, Guoqing Tang, Yanzhi Jiang, Mingzhou Li, Lei Chen, Lin Bai, Surong Shuai, Jinyong Wang, Xuewei Li, Kun Liao, Shunhua Zhang, Li Zhu

**Affiliations:** 1 College of Animal Science and Technology, Sichuan Agricultural University, Chengdu 611130, China; shenlinyuan0815@163.com (L.S.); 18299095425@139.com (M.G.); wupie@163.com (Q.T.); tyq003@163.com (G.T.); mingzhou.li@163.com (M.L.); chenlei815918@163.com (L.C.); blin16@126.com (L.B.); srshuai@sohu.com (S.S.); lixuewei9125@126.com (X.L.); 2 College of Life and Science, Sichuan Agricultural University, Chengdu 611130, China; jiangyz04@163.com; 3 Institute of Swine Science, Chongqing Academy of Animal Sciences, Chongqing 402460, China; kingyou@vip.sina.com; 4 Bashan Animal Husbandry Technology Co., LTD, Tongjiang 636700, China; 18181386956@163.com

**Keywords:** long non-coding RNAs, oxidative muscle, glycolytic muscles, pig

## Abstract

The biochemical and functional differences between oxidative and glycolytic muscles could affect human muscle health and animal meat quality. However, present understanding of the epigenetic regulation with respect to lncRNAs and circRNAs is rudimentary. Here, porcine oxidative and glycolytic skeletal muscles, which were at the growth curve inflection point, were sampled to survey variant global expression of lncRNAs and circRNAs using RNA-seq. A total of 4046 lncRNAs were identified, including 911 differentially expressed lncRNAs (*p* < 0.05). The *cis*-regulatory analysis identified target genes that were enriched for specific GO terms and pathways (*p* < 0.05), including the oxidation-reduction process, glycolytic process, and fatty acid metabolic. All these were closely related to different phenotypes between oxidative and glycolytic muscles. Additionally, 810 circRNAs were identified, of which 137 were differentially expressed (*p* < 0.05). Interestingly, some circRNA-miRNA-mRNA networks were found, which were closely linked to muscle fiber-type switching and mitochondria biogenesis in muscles. Furthermore, 44.69%, 39.19%, and 54.01% of differentially expressed mRNAs, lncRNAs, and circRNAs respectively were significantly enriched in pig quantitative trait loci (QTL) regions for growth and meat quality traits. This study reveals a mass of candidate lncRNAs and circRNAs involved in muscle physiological functions, which may improve understanding of muscle metabolism and development from an epigenetic perspective.

## 1. Introduction

Skeletal muscle accounts for approximately 40% of the total body mass in mammals [1], and is heterogeneous in nature. Based on the heterogeneous characteristic of movement rates and metabolic types, skeletal muscle fibers are broadly classified into different isoforms. For example, fast-twitch muscles, characterized by glycolytic metabolism and transient contractions, are generally referred to as white muscles. In contrast, slow-twitch muscles with more myoglobin, oxidative enzymes, and enduring of continuous movement, are called red muscles [2]. Regarding movement, the heterogeneity of muscle fibers allows muscles to work for various motor tasks, such as body posture, locomotion, jumping and kicking [3]. In terms of metabolism, fiber type diversification may also be linked to whole body metabolism of plasma glucose, which agrees with previous studies that have shown that slow-oxidative muscle fibers were more powerful than fast-glycolytic fibers in absorbing glucose from blood [4]. Increasing the composition ratio of fast-glycolytic fibers may contribute to insulin resistance and type-2 diabetes [5,6]. Other differential phenotypes of internal structures (mitochondria volume, myoglobin, and lipid content) and extracellular surroundings (capillary density and arteriole responsiveness) in heterogeneous muscle fibers also contribute to differential metabolic activities, such as ATP consumption and regeneration [7,8], gluconeogenesis [9,10], lactate, and fatty acid transport [11,12].

The diversity in contraction physiology and metabolic activity of different fiber types results not only in widely biological functions, but also in differential susceptibility to certain muscle diseases [13]. For example, Webster et al. (1988) showed that glycolytic fibers were much more susceptible to degeneration than oxidative fibers in duchenne muscular dystrophy (DMD) patients [14], which also agreed with the fact that glycolytic fibers were more susceptible to dystrophy characterized in facioscapulohumeral muscular dystrophy (FSHD), congenital fiber-type disproportion (CFTD), myotonic dystrophies type 2 (DM2), and aging-induced sarcopenia patients [15,16,17,18]. Although it’s well known that muscle diseases often affect particular fiber types, the underlying molecular mechanisms involved with the phenotype remain largely unclear. Furthermore, heterogeneous muscle fibers are also closely related to the process of muscle converting to meat in animals, thereby influencing the meat quality. For example, the composition ratio of glycolytic muscle fiber is negatively associated with pH_45_ value (45 min postmortem), and oxidative muscle fiber is negatively associated with drip loss [19]. It’s well known that the diversity of muscle metabolic activities is closely related with human muscle diseases and animal meat quality. However, different muscles have consistent DNA sequences because they originate from the same zygote. Therefore, epigenetic regulation is the main mechanism underlying different phenotypes [20]. With the development of high throughput sequencing techniques, more and more sequencing methods are being used to explore the genetic mechanisms underlying diverse biological functions between oxidative and glycolytic muscles. For example, mRNA transcriptome, microRNA transcriptome, and DNA methylome were used to identify novel regulatory factors which are involved with the diverse phenotypes [20,21,22]. Moreover, recent reports have shown that long noncoding RNAs (lncRNAs) and circular RNAs (circRNAs) may play important roles in various biological processes, including cell differentiation and development, and also in some diseases [23,24,25,26]. However, little is known about the biological functions and molecular mechanisms of lncRNAs and circRNAs in regulating diverse biological characteristics between oxidative and glycolytic muscles. Our previous study found that muscle tissues have maximum global transcriptional activity at the age of growth curve inflection point (maximum metabolism rate) [27]. Therefore, in this study, to explore the maximum non-coding RNA transcriptome difference between oxidative and glycolytic muscles, pigs were used as a model to identify distinct lncRNAs and circRNAs between oxidative and glycolytic muscles at growth curve inflection point. It’s well known that pigs are an ideal biomedical model for metabolic studies because their physiology, metabolism, and organ sizes are similar to humans [28]. We believe this study will uncover enormous novel signaling pathways related to biochemical and functional differences between oxidative and glycolytic muscles, and will also provide important insights into muscle disease pathologies and potential treatments.

## 2. Results and Discussion

### 2.1. Sigmoidal Growth Curve of Qingyu Pigs

**Figure 1:**
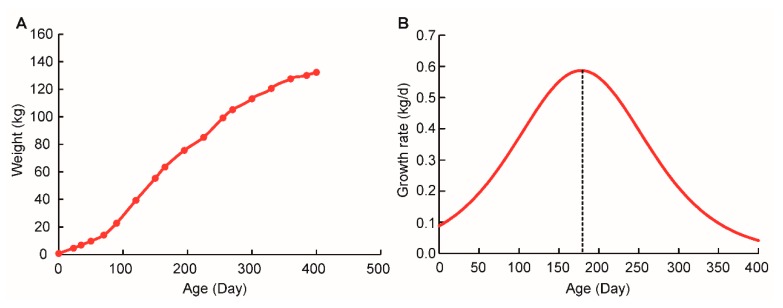
Growth curve of Qingyu pigs. (**A**) Sigmoidal curve fitted using logistic model, *n* = 126. (**B**) Daily gain of Qingyu pigs fitted by using a logistic equation, *n* = 126.

In a classical growth development sigmoidal curve, the mammalian postnatal growth rate continues to increase slowly until it reaches the maximum at growth inflection point; it then decreases asymptotically [29]. Our previous study has shown that pigs have higher global transcription and translation activities at inflection point than that at non-inflection point [27]. Therefore, to explore the maximum diversity of global transcriptome between oxidative and glycolytic skeletal muscles, logistic function was used to fit the growth curve of Qingyu pigs and predict the growth inflection point [30]. According to the growth data from 126 female Qingyu pigs ranging from birth to 400 days of age, we found that logistic models had a good fit with a typical sigmoidal curve (Figure 1 and Supplementary Table S1); the goodness of fit reached 0.97 (Supplementary Table S1). As shown in Figure 1A, Qingyu pigs reached the inflection point of the growth curve at the age of 177.96 days (Supplementary Table S2). Compared to commercialized pig breeds (such as Durocs), Qingyu pigs reached their inflection point later, but were similar with other Chinese native breeds [29,31]. This agreed with the fact that native pig breeds have a lack of long-term artificial selection for the purpose of improving growth rate.

### 2.2. Differences in Phenotypic Traits between Oxidative and Glycolytic Skeletal Muscles

**Figure 2:**
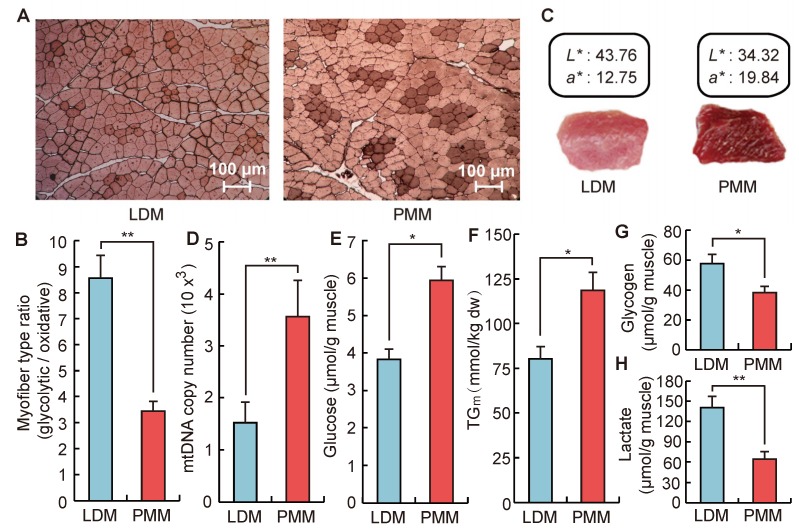
Different phenotypic indexes between oxidative (PMM) and glycolytic (LDM) skeletal muscles. (**A**) ATPase staining identifies slow-twitch (Type I) muscle fiber (dark color) and fast-twitch (Type II) muscle fiber (light color) in LDM and PMM, *n* = 8. (**B**) The muscle fiber types ratio (Fast/Slow) between LDM and PMM, *n* = 8. (**C**) The color of fresh samples of LDM and PMM, *n* = 8. (**D**) Mitochondrial DNA copy number per cell in LDM and PMM, *n* = 8. (**E**–**H**) The concentration of glucose, triglyceride, lactate, and glycogen in LDM and PMM, respectively, *n* = 8. Data are means ± SEM. Statistical significance was calculated by Student’s *t*-test. Significant difference levels: * *p* < 0.05, ** *p* < 0.01.

Based on the revealed inflection point, oxidative and glycolytic skeletal muscles of Qingyu pigs were sampled at 178 days of age for further phenotypes and transcriptome analysis. As shown in Figure 2A,B, the composition of oxidative myofiber in *psoas major* muscle (PMM) was approximately three times that of *longissimus dorsi* muscle (LDM). This result agreed with a previous report that LDM and PMM were typical glycolytic and oxidative muscle tissue, respectively [32]. In Figure 2C, the color of LDM had a higher L* (measure of lightness) and a lower a* (measure of redness) than that of PMM; this agrees with previous studies showing that oxidative fibers contained approximately 50% more myoglobin than glycolytic fibers [33]. Moreover, compared with PMM, LDM had a lower mitochondrial DNA content and a lower glucose and triglyceride concentration (Figure 2D–F). LDM, however, had a higher glycogen and lactic acid content (Figure 2G,H). Those phenotypes were closely related to different mechanisms of glycolysis and oxidative phosphorylation between oxidative and glycolytic muscles. Previous studies have shown that oxidative muscles produced ATP by oxidative mitochondrial processes, and contributed to the sustainability of muscle contractile activity for a long time without showing fatigue. However, glycolytic muscles were easily fatigued because they relied upon glycolytic processes to generate ATP rapidly, which was responsible for glycogen and glucose metabolism leading to lactate accumulation in muscles [2]. Additionally, the diverse lipid contents and the capacity of plasma glucose uptake in different myofiber types were also linked to the development of obesity and type-2 diabetes [34,35]. Therefore, considering the consistent DNA sequence of oxidative and glycolytic muscles from the same donor, the diversity ofmetabolic properties may be significantly regulated by epigenetic regulatory factors, such as lncRNAs and circRNAs.

### 2.3. Identification of mRNAs, lncRNAs, and cricRNAs in Oxidative and Glycolytic Skeletal Muscles

**Figure 3:**
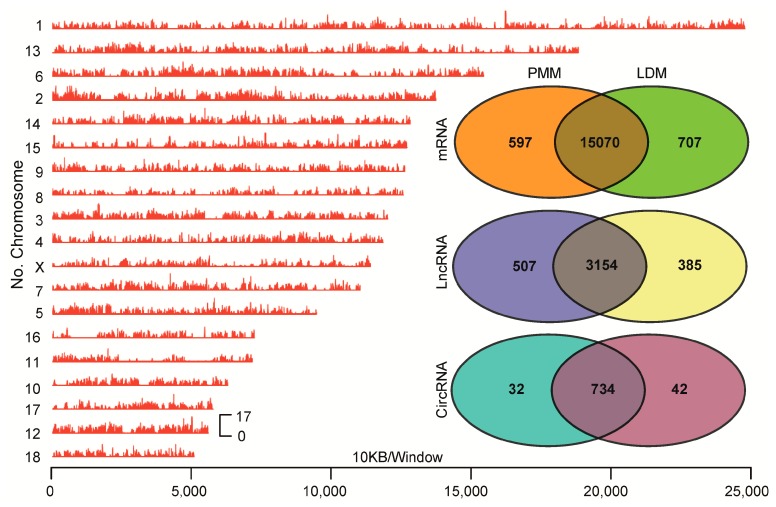
The distribution of sequenced reads and expressed transcripts in muscles. The left panel represented genome-wide distribution of sequenced reads in pig chromosomes. The right panel of Venn diagrams represented all the numbers of expressed mRNAs, lncRNAs, and circRNAs between LDM and PMM.

As shown in Table 1, a total of 540,198,540 raw reads (81.02 gigabases) were generated from the 6 cDNA libraries, representing approximately 34× coverage of the complete pig genome (*Sus scrofa* 11.1). After discarding adaptor sequences and low-quality reads, approximately 58.98–63.15% of all reads were uniquely and symmetrically mapped to the pig chromosomes (Figure 3): 29.68–31.76% and 29.30–31.39% of all reads were mapped to sense strand and antisense strand, respectively (Supplementary Table S4). Meanwhile, 0.72–1.10% of back-spliced junction reads were obtained for further circRNA identification (Supplementary Table S3). In this study, only the transcript expressed in all three biological replicates can be classified as the kind of expressed transcript, and used for subsequent analysis. Consequently, 15,667 and 15,777 known mRNA transcripts expressed in oxidative and glycolytic muscle were identified, respectively. A total of 3663 and 3539 novel lncRNA transcripts were identified from oxidative and glycolytic muscle, respectively. A total of 766 and 776 novel circRNA transcripts were identified from oxidative and glycolytic muscle, respectively (Figure 3).

**Figure 4:**
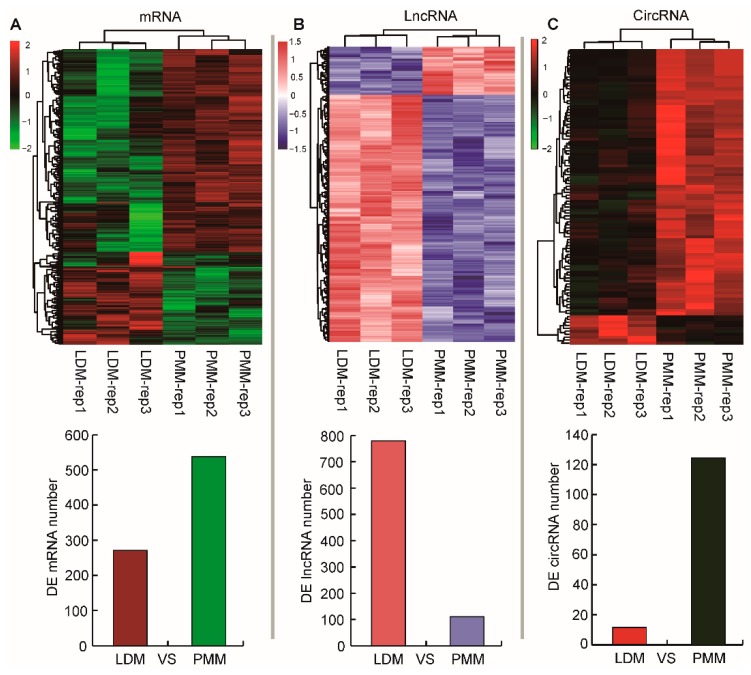
Hierarchical clustering analysis for differentially expressed (DE) mRNAs (**A**), lncRNAs (**B**), and circRNAs (**C**) between LDM and PMM.

To further explore the global transcriptional changes, a total of 810 differentially expressed mRNAs were identified between oxidative and glycolytic skeletal muscles, including 265 highly expressed genes in LDM and 545 highly expressed genes in PMM (Figure 4A). Also, 911 differentially expressed lncRNAs were identified from oxidative and glycolytic muscles, including 789 highly expressed lncRNAs in LDM, and 122 highly expressed lncRNAs in PMM (Figure 4B). In addition, 137 differentially expressed circRNAs between oxidative and glycolytic muscles were identified, comprising 12 highly expressed circRNAs in LDM, and 125 highly expressed cricRNAs in PMM (Figure 4C). The heat map of hierarchical clustering analysis indicated that all differentially expressed mRNAs, lncRNAs, and circRNAs were highly reproducible (Figure 4). These results prove the high reproducibility and reliability of transcriptome profiling performed in the present study.

### 2.4. Function Enrichment Analysis of Nearest Neighbor Genes of Differentially Expressed lncRNAs

**Figure 5:**
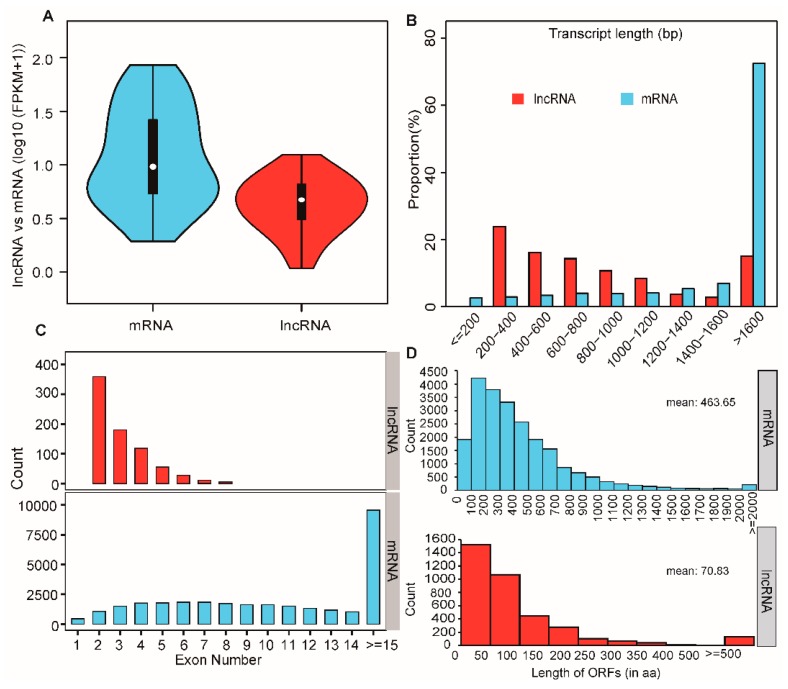
Comparison of genomic features of mRNAs and lncRNAs. (**A**) Expression levels of total mRNAs and lncRNAs using log_10_ (FPKM + 1). (**B**) The transcript length of mRNAs and lncRNAs. The x axis represents the length of transcripts, and the y axis represents the composing proportion. (**C**) Distribution of exon numbers of mRNAs and lncRNAs. (**D**) Distribution of open reading frames (ORFs) lengths in mRNAs and lncRNAs.

In the present study, a total of 4046 lncRNAs were obtained from pig skeletal muscles, most of which were defined as intergenic lncRNAs (80.48%) (Supplementary Table S4 and Figure S1). To explore differential features between lncRNAs and mRNAs, the average expression levels of 4046 lncRNAs and 16,374 mRNAs were estimated as log_10_ (FPKM + 1). This showed that the average expression level of lncRNAs was lower than that in mRNAs (Figure 5A), which was similar to previous findings in pig adipose tissues [36]. In addition, we found that lncRNAs with an average length of 754 bp and 2.8 exons were shorter than mRNAs with an average length of 2453 bp and 8.9 exons (Figure 5B,C). This was in accordance with previous studies showing that lncRNAs were shorter in length and had fewer exons than protein coding transcripts [37,38]. Furthermore, it revealed that the average length of the predicted ORFs in the lncRNAs was 70.83 bp. However, the average length of mRNA ORFs was 463.65 bp (Figure 5D), which agreed with a previous study reported the typical characterization of lncRNAs [39]. These results suggest that the identified lncRNAs in the present study are highly reliable.

**Figure 6:**
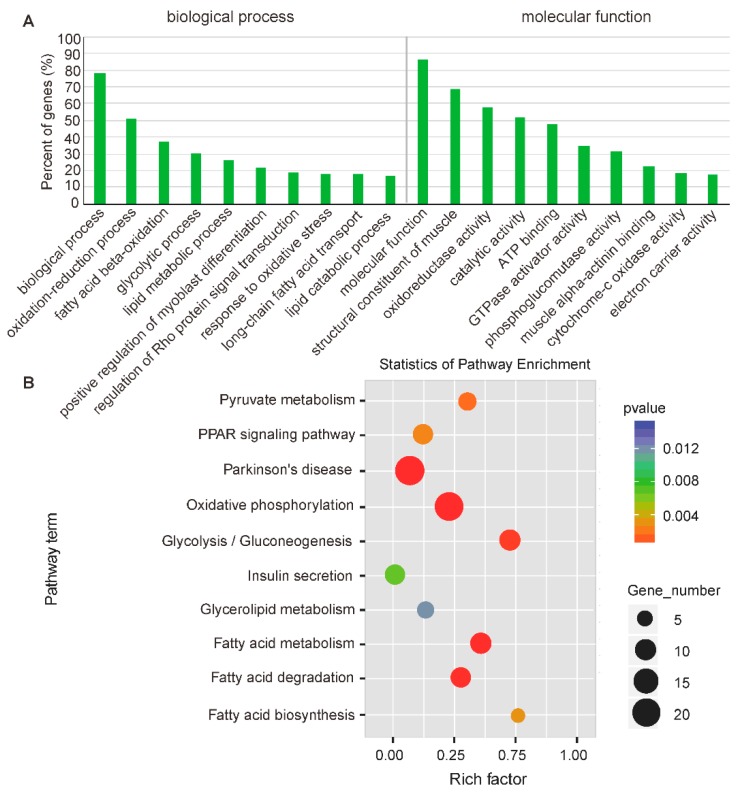
Functional enrichment analysis of differentially expressed *cis* lncRNA target genes between LDM and PMM. (**A**) Gene Ontology (GO) terms and (**B**) KEGG pathways enriched for neighboring target genes of DE lncRNAs (*cis*-regulation). Benjamin-corrected modified Fisher’s exact test was used to calculate the *p* values.

Lots of research projects have shown that lncRNAs may act in *cis*-regulation and affect the gene expression of their chromosomal neighborhood within 10 kb of upstream and downstream [40,41]. Therefore, to investigate the possible functions of lncRNAs on muscle physiology, protein-coding genes were searched in 10-kb upstream and downstream of all the identified differentially expressed lncRNAs. We found that 453 lncRNAs were significantly differentially expressed between LDM and PMM, which were transcribed closely to (<10 kb) 481 differentially expressed mRNAs. Gene Ontology (GO) analysis of the *cis* lncRNA target genes was performed to explore their functions. We found that 75 GO terms were significantly enriched (*p* < 0.05), and the top ten enriched terms included “oxidation-reduction process”, “glycolytic process”, “fatty acid metabolic process”, and “GTPase activator activity” (Figure 6A). Previous studies have shown that glycolytic muscles have a lower oxidative capacity, ATP synthesis efficiency, and mitochondria content than oxidative muscles [2,42]. This agrees with the results of enriched GO terms related to “ATP binding”, “response to oxidative stress”, “oxidoreductase activity”, and “electron carrier activity” in the present study (Figure 6A). Interestingly, we also found genes *pyruvate kinase isoenzyme M2* (*PKM2*) and *hexokinase 2* (*HK2*), both of which were annotated with a glycolytic process-related GO term (GO:0006096). *PKM2* and *HK2* are the predominant genes encoding rate-limiting enzymes that catalyze the glycolysis reaction by degrading glycogen [42]. All these also were supported by the phenotypes of LDM, which have a higher glycogen and lactate content than PMM (Figure 1G,H). These results suggest that glycolytic potential in muscles may be regulated by actions of lncRNAs on these neighboring protein-coding genes. Additionally, our previous studies found that GTPase superfamily (Ras, Rac, Rho, Rab, and Ran) regulated different phenotypic traits between oxidative and glycolytic skeletal muscles through signaling cascades and feedback loop regulation [20]. Here, we found a significantly enriched GO term related to “Rho protein signal transduction” (GO:0035023) and “GTPase activator activity” (GO:0005096) (Figure 6A).

Potential targets of lncRNAs in *cis*-regulatory relationships were predicted using pathway analysis, which showed that the top ten Kyoto Encyclopedia of Genes and Genomes (KEGG) pathways were related to “fatty acid metabolism”, “PPAR signaling pathway”, “oxidative phosphorylation”, and “glycolysis” (Figure 6B). Previous studies have found that triglyceride concentration (7 mM vs. 4.2 mM) and lipid droplets (0.5% vs. 0.1%) in slow (oxidative) fibers are lower than that in fast (glycolytic) fibers [43,44]. Here, we found genes, including fatty acid translocase (FAT/CD36) and fatty acid-transport protein 6 (FATP6), were annotated to “fatty acid biosynthesis” pathway (ko.00061). These two genes were proven to regulate the process of transferring fatty acid from serum into muscle [45]. The PPAR signaling pathway was involved in the regulation of *PPARβ-PGC1α* signaling cascade, which is the most crucial network responsible for fiber-type switching [34]. Therefore, these findings indicate that lncRNAs play an important role in *cis*-regulating different phenotypic traits between oxidative and glycolytic skeletal muscles.

### 2.5. Function Analysis of Differentially Expressed circRNAs between Oxidative and Glycolytic Muscles

**Figure 7:**
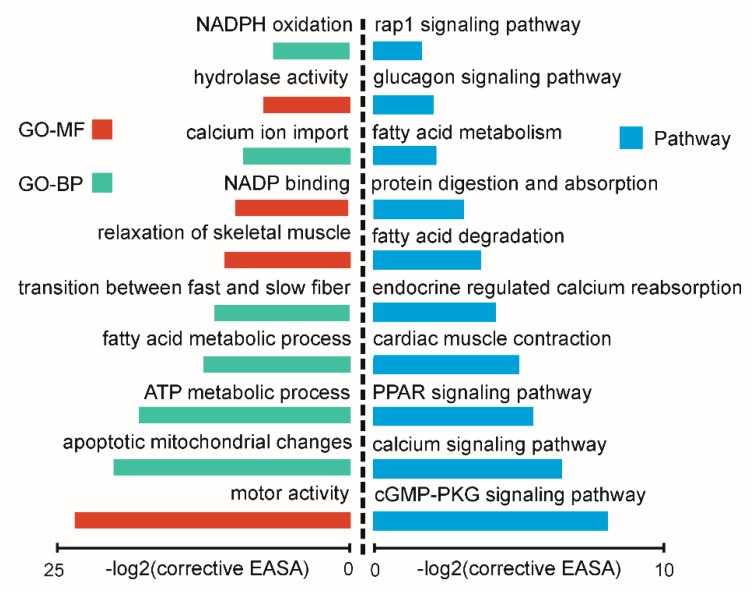
The top ten GO terms and KEGG annotations for host genes of differentially expressed circRNAs. The EASE score, which indicated the significance of comparisons, was calculated by Benjamin-corrected modified Fisher’s exact test.

Recently, many studies have shown that circRNA expressions could be regulated by linear transcripts derived from host genes [46,47]. Therefore, to explore the mechanism of circRNAs in regulating muscle physiology, circRNA host genes were used to perform a function enrichment analysis using DAVID software. The top ten significantly over-represented gene ontology (GO) terms were related to “motor activity”, “transition between fast and slow fiber”, and “ATP metabolic process” (Figure 7). The top ten significantly enriched KEGG pathway were related to “PPAR signaling”, “calcium signaling”, and “fatty acid metabolism” (Figure 7). These results were highly consistent with the *cis*-regulatory function of lncRNAs, and also strongly related with different phenotypic traits between LDM and PMM. This implied that circRNA host genes may have important biological functions by regulating circRNA expressions. Interestingly, we found that differentially expressed *circRNA153*, *circRNA41*, and *circRNA69* were transcribed from *MYH1* (coding MyHC-2X protein), *MYH7* (coding MyHC-β protein), and *MYH2* (coding MyHC-2A protein) gene, respectively. These results suggest that the three circRNAs may play important roles in regulating fiber type heterogeneity in muscles.

**Figure 8:**
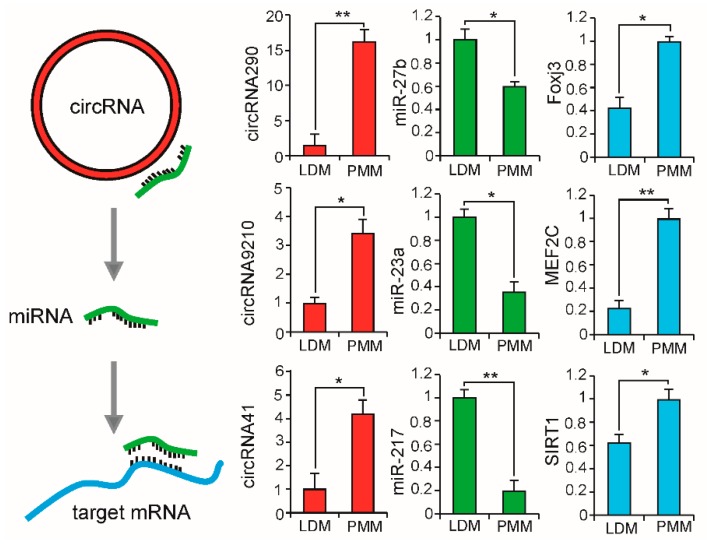
The differentially expressed circRNA-miRNA-mRNA network between LDM and PMM. qPCR was used to detect expression levels of circRNAs, miRNAs, and mRNAs. All data are presented as means ± SEM. Student’s *t*-test used to calculate statistical significance (* *p* < 0.05, ** *p* < 0.01), *n* = 3.

Previous researchers have found circRNAs served as miRNA sponges which could negatively regulate the expression levels of miRNAs by competing endogenous RNA (ceRNA) networks [48,49]. Here, potential targets of circRNAs were predicted based on the complementary seed sequences of miRNAs (2–8 bp). The top ten differentially expressed circRNAs between LDM and PMM were shown in Table 2, and only one circRNA was up-regulated in LDM. On the basis of bioinformatics analysis, two of the ten cicrRNAs, *circRNA290* and *circRNA9210*, were found to share binding sites with seed sequences of *miR-23a* and *miR-27b*. Interestingly, previous studies have shown *miR-23a* and *miR-27b* could reduce the composition ratio of slow myosin heavy chain isoform by targeting *myocyte enhancer factor 2C* (*MEF2C*) and *forkhead box j3* (*Foxj3*), respectively [50,51]. In consistent with previous findings, *circRNA290* and *circRNA9210*, which targeted with *miR-23a* and *miR-27b* and also decreased the expression of *miR-23a* and *miR-27b*, resulting in increasing expressions of target genes (*MEF2C* and *Foxj3*) (Figure 8), were highly expressed in PMM. Interestingly, the expression pattern of these genes agreed with the phenotype of PMM, which has a higher slow-oxidative myofiber proportion than LDM. These results suggest that *circRNA290-miR27b-Foxj3* and *circRNA9210-miR-23a-MEF2C* networks have a potential role in regulating fiber-type switching. Additionally, previous study has found *SIRT1* (target of *miR-217* [52]) could induce mitochondrial biogenesis by deacetylation of *PGC-1α* [53]. As shown in Figure 8, the expression pattern of *circRNA41-miR-217-SIRT1* network in LDM and PMM was consistent with the phenotype of high mitochondria content in PMM (Figure 2D). Overall, these findings indicate that circRNAs are a critical factor in regulating physiological function in muscles by competing endogenous miRNAs.

### 2.6. QTL Enrichment Analysis of Differentially Expressed Transcripts

Previous studies have reported that heterogeneous muscle fibers were closely related to animal economic traits, such as meat quality and growing development [54,55]. To explore the differentially expressed transcripts related to pig growth and meat quality traits, an enrichment analysis was performed with these differentially expressed mRNAs, lncRNAs, and circRNAs though BLAST analysis with a high confidence and narrowed (<2 Mb) QTL interval. As shown in Table 3, 362 (44.69%) differentially expressed mRNAs had a significant enrichment in QTL regions. For example, CPT2 was localized to chromosome 6 within the interval of QTLs for saturated fatty acid content. FNDC1 was localized to the interval of QTLs for drip loss (Supplementary Table S5). It was found that 357 (39.19%) differentially expressed lncRNAs had a significant enrichment in QTL regions. Most of these enriched QTL regions were related to meat quality traits. One such gene was lncRNA4177, which was localized to chromosome 13 within the interval of QTLs for water holding capacity, pH 45 min post-mortem, average daily gain, and average backfat thickness (Supplementary Table S6). Furthermore, 74 (54.01%) differentially expressed circRNAs were identified to enrich in QTL regions related to growth and meat quality traits. For example, circRNA227 was localized to chromosome 6 within the interval of QTLs for intramuscular fat content and loin muscle area. CircRNA11553 was localized to the interval of QTLs for mean corpuscular hemoglobin content (Supplementary Table S7). These observed results were consistent with the variant phenotypic characteristics between oxidative and glycolytic skeletal muscles.

### 2.7. Validation of differentially expressed lncRNAs and circRNAs

**Figure 9:**
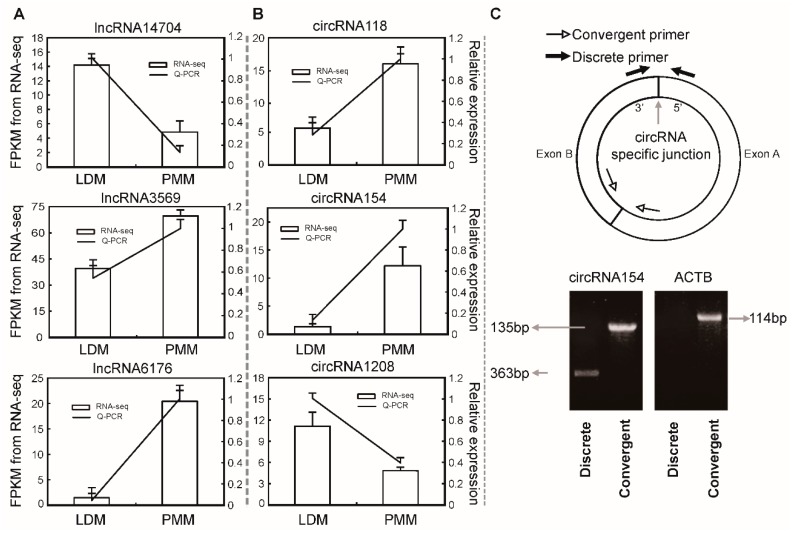
Using qPCR to validate RNA-seq data. (**A**) RNA-seq and q-PCR data of expression levels of 3 randomly selected lncRNAs, *n* = 3. (**B**) RNA-seq and q-PCR data of expression levels of 3 randomly selected circRNAs, *n* = 3. (**C**) The illustration of qPCR primers for identifying circRNAs, *n* = 3.

To further validate the high throughput sequencing results in this study, three differentially expressed lncRNAs and circRNAs were randomly selected to detect their expression patterns between LDM and PMM by qPCR. As shown in Figure 9A,B, the qPCR results indicated that the expression patterns of these lncRNAs and circRNAs were highly consistent between two methods. In addition, convergent and discrete primers were designed to validate the identified circRNAs by bioinformatics analysis. As shown in Figure 9C, only the discrete primers could amplify PCR products from circRNA transcripts. For example, the circRNA154 amplified its junction region (363 bp) using discrete qPCR primers, but no PCR product was amplified from linear transcripts (such as ACTB). These results suggest that the RNA-Seq data and bioinformatics analysis pipeline used in the present study have a high reliability.

## 3. Materials and Methods

### 3.1. Ethics Statement

All the animal care and sample collection were approved by the Institutional Animal Care and Use Committee of the College of Animal Science and Technology of Sichuan Agricultural University, Sichuan, China, under permit No. DKY-B20131403 (Ministry of Science and Technology, China, approved in 15 June 2004).

### 3.2. Animal Materials and Tissue Collection

In this study, a total of 126 female Qingyu pigs (Chinese native breeds, Bashan animal husbandry technology co., LTD, Tongjiang, Sichuan, China) were fed from birth to 400 days old. The data of growth traits at 19 time points were measured to calculate the inflection point of growth curve by Logistic non-linear models [30]. In all stages, pigs had ad libitum access to feed (formulas shown in Supplementary Table S8) and water, and were housed in the same farm environment. Meanwhile, another 8 female pigs at inflection point (178 days old) were used to harvest *longissimus dorsi* muscle (LDM, glycolytic skeletal muscle) samples from the core of the adjacent to the last rib, and *psoas major* muscle (PMM, oxidative skeletal muscle) samples were obtained from the core of the adjacent to the last 4 ribs. All samples were used for phenotypic traits analysis (*n* = 8), and 3 out of 8 samples were randomly selected for lncRNA and circRNA transcriptome analysis. The 8 pigs were kept off feed but given free access to water for 24 h, and then electrically stunned, exsanguinated, scalded, and rinsed. All the samples were rapidly frozen in liquid nitrogen and stored at −80 °C until being used for RNA and DNA extraction. All pig feed formulas meet or exceed the National Research Council (NRC, 1998) recommendations for the different growth stages (Supplementary Table S8).

### 3.3. Growth Curve Models

The growth curve model used in this study was Wt = A/(1 + Be − kt) [56]. In this model, Wt represents the weight where the time point (*t*) was recorded; B is the growth curve line constant; k is taken as the inherent relative growth rate at the start; A represents the maximum size; A/2 represents the inflection point weight; lnB/k represents the inflection point age; kw/2 represents the maximum daily gain. R2 is the degree of fitting, which is calculated by the equation of R2 = 1 − RSE/RST. In the equation, RST represents the sum of squares of deviations RSE, and represents the residual sum of squares.

### 3.4. Measurement of Phenotypic Traits

The myofiber type ratio was measured using ATPase staining as previously described [57]. Briefly, ATPase reactions were performed after acid (pH 4.6 for 2 min) and alkaline (pH 9.4 for 5 min) pre-incubations, respectively. After staining, the fast-glycolytic fibers showed heavy coloring, whereas slow-oxidative fibers showed light coloring. The myofiber type ratio was measured by the area of glycolytic fibers divided by the area of oxidative fibers in 100 randomly chosen fields of vision. The chosen fields were examined on serial cryosections, which were produced from 8 individuals. Mean area of glycolytic fibers and oxidative fibers was deemed as the average area of myofiber. To detect other metabolites, about 500 mg of frozen muscle sample was weighed and homogenized in 500 mL of 0.9% saline, and then centrifuged at 4200× *g* for 10 min at 4 °C. The supernatant was diluted 50 times for measuring Gly (glycogen, E07G0023), Glu (glucose, E07G0030), and LA (lactate, E07L0004) content by using standard commercial kits from BlueGene Biotech Co., Ltd. (Shanghai, China). Optical density (OD) at 450 nm was immediately measured utilizing an ELISA microplate reader. Consequently, a calibration curve was constructed using OD values corresponding to each concentration of the standard. To measure the triglyceride content in muscles, samples were prepared according to previous descriptions [58]. The triglyceride concentration determined by commercial kits (E07T0018, BlueGene Biotech Co., Shanghai, China) following the manufacturer’s instructions. Besides, muscle color was measured with a Minolta chromameter (CR-300, Minolta Cam-era Co., Osaka, Japan) at 45 min postmortem. The average of triplicate measurements was recorded, and the results were expressed as CIELAB (Commission International de l’eclairage) lightness (L*) and redness (a*) values. All the phenotypic traits were measured from 8 individual pigs at the age of 178 days (*n* = 8).

### 3.5. Transcriptome Library Construction and Sequencing

According to our previous study [59], total RNA was extracted from PMM and LDM using TRIZOL (Invitrogen, Carlsbad, CA, USA), and further purified with RNeasy column (Qiagen, Valencia, CA, USA) according to the manufacturer’s protocol. Total DNA was isolated from LDM and PMM for the measurement of mtDNA copy number by using the DNeasy Blood & Tissue Kit (Qiagen, USA). RNA integrity and concentration were analyzed with the Bioanalyzer 2100 (Agilent Technologies, Foster City, CA, USA). Three biological replicates in each group were used to construct transcriptome library. Briefly, only the RNA samples that had RNA Integrity Number (RIN) scores higher than 8 were used for further library construction. A total of 3 μg RNA per sample was used as input material for RNA sample preparation. mRNAs and non-coding RNAs were enriched by removing rRNAs from the total RNA with a commercial kit (Epicentre, Madison, WI, USA). By using the fragmentation buffer (Ambion, Foster City, CA, USA), mRNAs and non-coding RNAs were fragmented into short fragments (about 200–700 bp), then the first-strand cDNA was synthesized by random hexamer-primer using the fragments as templates. Buffer, dNTPs, RNase H, and DNA polymerase I were added to synthesize the second strand cDNA. The double strand cDNA was purified with QiaQuick PCR extraction kit (Qiagen, USA) and then used for end-polishing. Sequencing adapters were ligated to the fragments; then, the second strand was degraded using UNG (Uracil-N-Glycosylase) finally. The fragments were purified by Agarose gel electrophoresis and enriched by PCR amplification. The library products were ready for sequencing analysis via Illunima HiSeqTM 4000, and 150 bp long paired-end reads were generated. All the high-throughput sequencing data have been deposited in NCBI’s Gene Expression Omnibus under GEO Series accession numbers GSE119137.

### 3.6. Identification of mRNAs, lncRNAs, and circRNAs

As in our previous study [59], the clean data were obtained by removing reads containing adapters, reads containing over 10 % of poly (N), and low-quality reads (>50 % of the bases had Phred quality scores ≤ 10) from the raw data. All the downstream analyses were based on high quality clean data. Pig reference genome and gene annotation files were downloaded from NCBI database (*Sscrofa*11.1, NCBI). Paired-end clean reads were aligned to the reference genome using Tophat (version 2.1.1, download site: http://ccb.jhu.edu/software/tophat/index.shtml) package, and parameters were set as default. The mapped reads from each library were assembled with Cufflinks (version 2.1.1) to construct and identify mRNA transcripts. Next, the data analysis was performed by filtering the assembled novel transcripts from the different libraries to obtain putative lncRNAs following the steps in the pipeline as follows. First, transcripts that were shorter than 200 nt in length, containing fewer than 2 exons and fewer than three reads were excluded. Next, using the Coding-Non-Coding Index (CNCI) [60] and Coding Potential Calculator (CPC) [61] to evaluate the coding potential of the filtered transcripts. A transcript with a CNCI value lower than 0 and a CPC value lower than -1 was taken to be an lncRNA. In this study, the find_circ software was used to identify circRNAs. Briefly, the unmapped back-spliced junction reads were used to extend the anchor sequences by find_circ software with default parameters [62]. Besides, identified circRNAs that expressed in at least two samples were used for further analysis. The expression levels of mRNAs, lncRNAs, and circRNAs were quantified by the value of fragments per kilobase of exon model per million fragments mapped (FPKM). Differentially expressed mRNAs, lncRNAs, and cricRNAs were filtrated by Cuffdiff software with parameters of *p* value < 0.05 and |log_2_ (fold change)| ≥ 1.

### 3.7. Functional Enrichment Analysis

LncRNAs have been shown to regulate the expression of neighboring genes (*cis*-acting regulation) [63]. Therefore, coding genes were searched from 10-kb upstream and downstream of all the identified differentially expressed lncRNAs; then, their functional roles were predicted as follows. The names of the neighboring genes were used to form a gene list to input into DAVID software (DAVID 6.8) for Gene Ontology (GO) analysis according to our previous study [59]. A Kyoto Encyclopedia of Genes and Genomes (KEGG) enrichment analysis of the predicted target genes was performed with KOBAS software using a hyper-geometric distribution test. GO terms and KEGG pathways with Benjamin-corrected *p* value < 0.05 were considered to be significantly enriched. Finally, differentially expressed transcripts were used to map pig QTL regions according to previous descriptions [64]. QTL data were downloaded from the Pig Quantitative Trait Locus database (PigQTLdb: http://www.animalgenome.org/QTLdb/pig.html) website. When a transcript has an overlapping region with QTL (<2 Mb) regions (at least half length of the gene or the QTL), it was defined as a QTL transcript.

### 3.8. Quantitative RT-PCR

The quantitative RT-PCR (Q-PCR) was performed according to our previously described study [59]. Briefly, SYBR Green Real-time PCR Master Mix (TaKaRa, Dalian, China) was used to perform the reactions on CF96 Real-Time PCR Detection System (Bio-Rad). The 2^−ΔΔCt^ method was used to determine the relative mRNA abundance. *ACTB*, *TBP*, and *TOP2B* genes were simultaneously used as internal gene for normalization. The ratio of mitochondrial genes (*ATP6*, *COX2*, and *ND1*) to nuclear DNA single copy gene (*GCG*) within the same samples was used to calculate the relative mtDNA copy number. All reactions were performed in triplicate. All PCR primer sequences are shown in Supplementary Table S9.

### 3.9. Statistical Analyses

All data were analyzed using Student’s *t*-test analyses in SPSS (21.0 version, SPSS, Inc., Chicago, IL, USA). The significance level for the statistical analysis was *p* < 0.05. All data are presented in this paper as means ± standard error of the mean (SEM).

## 4. Conclusions

In summary, we conducted a genome-wide diversity analysis of lncRNA and circRNA expression in porcine oxidative and glycolytic skeletal muscles at growth curve inflection point (maximum growth rate). Nine hundred and eleven differentially expressed lncRNAs and 137 differentially expressed circRNAs were identified which were potentially associated with the glycolytic process, oxidation-reduction process, ATP metabolic, and the transition of fiber types. These enriched pathways were consistent with the different phenotypes between oxidative and glycolytic muscles, such as mitochondrial content, proportion of fiber types, and glycolytic potential. We also found that *circRNA290-miR27b-Foxj3* and *circRNA9210-miR-23a-MEF2C* were two competing endogenous networks which ware closely linked to muscle fiber-type switching. Interestingly, some differentially expressed transcripts were significantly enriched in pig QTL regions related to traits of meat quality and growing development. We believe that this study will contribute to the understanding of human muscle health and animal meat quality.

## Figures and Tables

**Table 1:** Summary of transcriptome data.

Sample	Raw Data (Reads)	Clean Data (Reads)	Data Size (Gb)	Clean Data Q20 (%)	GC Content (%)	rRNA Reads (%)
LDM_1	90164014	89626552	13.52	96.75	52.52	0.56
LDM_2	89601234	89357710	13.44	96.88	50.84	0.26
LDM_3	89028092	88835098	13.35	96.75	53.75	0.21
PMM_1	90586046	90269524	13.59	96.77	51.7	0.33
PMM_2	90321580	89987360	13.55	96.44	51.41	0.35
PMM_3	90497574	90141574	13.57	96.5	51.46	0.38

**Table 2:** The top ten differentially expressed circRNAs between LDM and PMM.

Accession	Fold Change (LDM/PMM)	Host Gene	Sponge miRNAs
*ciRNA290*	−16.97	*PAN2*	*miR-133a, miR-150, miR-221, miR-23a, miR-23b, miR-27a, miR-27b, miR-30b*
*circRNA8413*	−7.08	*AQP4*	*miR-9823*
*circRNA17323*	−5.94	*C11orf70*	*miR-9859*
*circRNA16211*	−5.38	*PANK1*	*miR-22, miR-339, miR-339, miR-676*
*circRNA5396*	−5.28	*PANK1*	*miR-22, miR-676*
*circRNA10701*	4.99	*SLC6A20*	*miR-2320, miR-4335, miR-885*
*ciRNA41*	−4.07	*MYH7*	*miR-217*
*ciRNA296*	−4.00	*CCDC88A*	*miR-421*
*circRNA12969*	−3.82	*CLIP4*	*miR-221, miR-23a, miR-23b, miR-27a,*
*circRNA9210*	−3.77	*CLIP4*	*miR-133a, miR-145, miR-150, miR-23a, miR-23b, miR-27a, miR-27b*

**Table 3:** Distribution of differentially expressed transcripts in chromosome and QTLs region.

Chromosome	DE Transcript Number in QTL Region/QTL Region Length (Mb)		
	mRNA	LncRNA	CircRNA
1	25/15.39	31/19.56	1/0.31
2	32/15.45	32/13.69	4/2.82
3	21/10.07	17/9.72	13/1.70
4	31/11.78	27/13.26	4/2.09
5	18/5.87	6/4.53	17/1.43
6	35/18.78	18/9.88	2/1.73
7	41/26.39	33/22.27	1/0.92
8	14/12.24	29/13.27	0/0
9	19/13.90	17/13.27	2/2.13
10	16/10.93	24/8.18	5/2.04
11	4/10.93	1/1.73	1/1.00
12	21/13.89	19/12.07	0/0
13	25/14.34	32/13.19	4/0
14	19/10.95	21/11.51	3/1.22
15	17/6.68	21/8.56	8/2.13
16	8/3.95	16/7.80	5/2.04
17	6/1.30	4/2.13	4/1.81
18	6/2.52	8/3.56	0/0
X	4/3.07	1/0.72	0/0
MT	0/0	0/0	0/0

The statistical significance was calculated by the χ^2^-test (*p* < 10^−4^). MT: mitochondria.

## Supplementary Materials

Supplementary materials can be found at https://www.mdpi.com/1422-0067/20/12/2855/s1.
