# Supplementary material for: Comprehensive Analysis of lncRNAs and circRNAs Reveals the Metabolic Specialization in Oxidative and Glycolytic Skeletal Muscles
Source: Int J Mol Sci. 2019 Jun 12;20(12):2855. doi: 10.3390/ijms20122855 (PMC6627206; doi:10.3390/ijms20122855)
Supplement: Supplementary file 1 [file ijms-20-02855-s001.zip › Supplementary_Material.pdf]

Supplementary Material

Comprehensive analysis of lncRNAs and circRNAs reflects the metabolic specialization in oxidative and glycolytic skeletal muscles

Linyuan Shen, Qianzi Tang, Guoqing Tang, Yanzhi Jiang, Mingzhou Li, Lei Chen, Lin Bai, Surong Shuai, Jinyong Wang, Xuewei Li, Kun Liao, Shunhua Zhang, & Li Zhu

Supplementary Table S1. The measured weight of Qingyu pigs

**Age**	**0 day**	**23 day**	**35 day**	**50 day**	**70 day**
BW	0.76±0.08	4.63±0.88	6.91±0.93	9.70±1.48	14.13±0.85
**Age**	**90 day**	**120 day**	**150 day**	**165 day**	**180 day**
BW	22.78±2.95	39.37±3.14	55.36±4.71	63.54±5.28	69.32±6.30
**Age**	**195 day**	**225 day**	**255 day**	**270 day**	**300 day**
BW	75.60±7.81	85.15±6.64	99.22±8.26	105.17±7.66	113.13±7.52
**Age**	**330 day**	**360 day**	**385 day**	**400 day**	
BW	128.32±9.75	137.94±7.78	143.04±7.49	146.65±6.26	

BW, body weight.

Supplementary Table S2. Fitting parameters of Logistic nonlinear curves

Model	k	a	b	R^2	Age (MGR)	Index (MGR)
Logistic	130.404	24.613	0.018	0.974	177.960	65.202

MGR, the point of maximum growth rate

Supplementary Table S3. Summary of transcriptome data

Sample	LDM_1	LDM_2	LDM_3	PMM_1	PMM_2	PMM_3
Valid reads	86930486	86411128	86581698	86844958	85596078	86113732
Mapped reads	71.91%	71.66%	75.01%	71.96%	72.09%	72.24%
Unique Mapped reads	61.51%	63.15%	58.98%	62.43%	61.42%	60.62%
Multi Mapped reads	10.40%	8.51%	16.03%	9.53%	10.67%	11.61%
PE Mapped reads	32.47%	32.38%	34.08%	32.54%	32.16%	32.51%
Mapped left reads	37.22%	36.79%	38.55%	37.22%	37.12%	37.39%
Mapped right reads	34.70%	34.87%	36.46%	34.74%	34.97%	34.84%
Reads map to sense strand	30.75%	31.76%	29.68%	31.22%	31.03%	30.46%
Reads map to antisense strand	30.76%	31.39%	29.30%	31.21%	30.38%	30.17%
Non-splice reads	36.06%	40.56%	33.66%	39.49%	38.88%	36.57%
Splice reads	25.45%	22.58%	25.32%	22.94%	22.54%	24.06%
Back-spliced junctions reads	0.85%	0.74%	1.10%	0.72%	0.98%	0.87%

Supplementary Table S4. Statistics of identified lncRNAs

Sample	"i"	"j"	"o"	"u"	"x"
LDM_1	293	13	39	1985	134
LDM_2	333	14	38	2329	141
LDM_3	262	15	39	1867	121
PMM_1	280	14	39	1837	133
PMM_2	261	15	39	1844	120
PMM_3	256	14	38	1675	124

J: Potentially novel isoform (fragment): at least one splice junction is shared with a reference transcript; I: A transfrag falling entirely within a reference intron; O: Generic exonic overlap with a reference transcript; U:Unknown, intergenic transcript; X:Exonic overlap with reference on the opposite strand

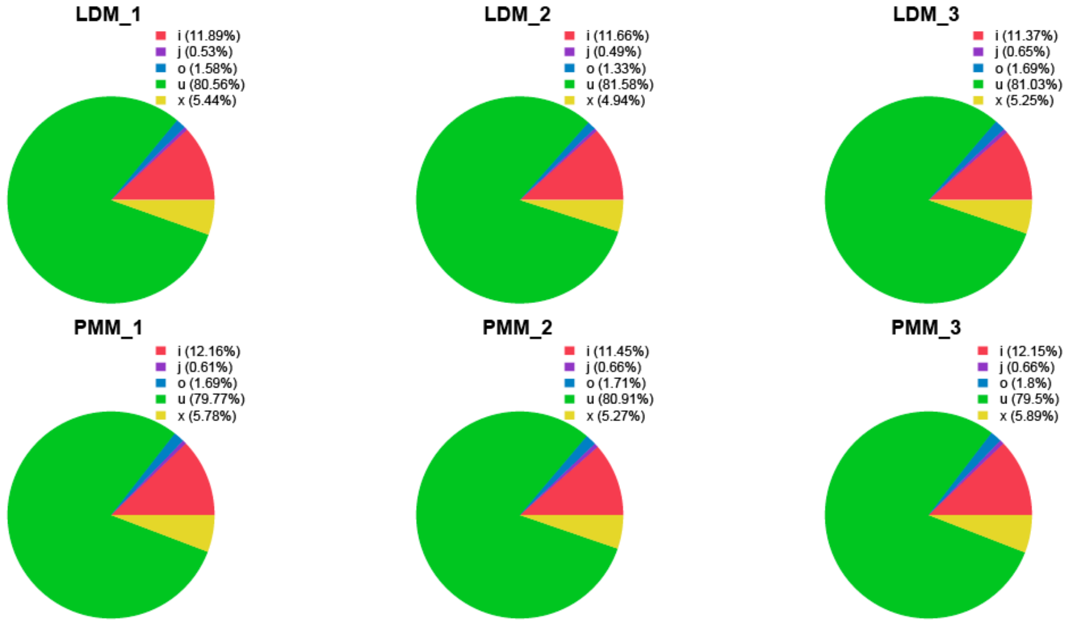

Supplementary Fig.S1. The type and distribution of identified lncRNAs. J: Potentially novel isoform (fragment): at least one splice junction is shared with a reference transcript; I:A transfrag falling entirely within a reference intron; O: Generic exonic overlap with a reference transcript; U:Unknown, intergenic transcript; X:Exonic overlap with reference on the opposite strand.

Supplementary Table S8. Ingredient composition and nutrient levels of expreimental diets (as fed basis,%)

Ingredients	7-11kg	11-25kg	25-50kg	50-75kg	>75kg
Corn grain	27.19	35.00	68.57	75.13	78.03
Expand corn	25.00	23.68	0.00	0.00	0.00
Soybean oil	2.50	1.80	3.00	3.00	3.00
Sucrose	2.00	2.00	2.00	0.00	0.00
Wheat bran	0.00	0.00	3.00	3.00	5.00
Whey powder	12.00	7.00	0.00	0.00	0.00
Soybean meal	8.00	12.00	18.20	15.50	10.90
Full-fat soybean	5.00	8.00	0.00	0.00	0.00
Soy protein concentrate	5.00	3.00	0.00	0.00	0.00
Whole milk powder	3.00	0.00	0.00	0.00	0.00
Fishmeal (CP 62.5 %)	4.00	3.00	2.00	0.00	0.00
Plasma protein powder	3.00	1.00	0.00	0.00	0.00
L-Lys·Hcl	0.45	0.46	0.42	0.44	0.40
DL-Met	0.15	0.16	0.14	0.12	0.10
L-Thr	0.15	0.16	0.14	0.15	0.14
L-Try	0.01	0.02	0.03	0.03	0.03
Choline chloride 50%	0.15	0.10	0.10	0.10	0.10
CaCO3	1.00	0.92	0.90	0.89	0.80
CaHPO4	0.10	0.40	0.70	0.84	0.70
NaCl	0.30	0.30	0.30	0.30	0.30
Premix[1]	1.00	1.00	0.50	0.50	0.50
Total	100.00	100.00	100.00	100.00	100.00
**Nutrient levels[2]**					
DE（Kcal/kg）	3.58	3.48	3.39	3.40	3.39
CP（%）	19.55	18.35	15.68	13.73	12.18
Ca（%）	0.79	0.71	0.66	0.59	0.52
STTD P（%）	0.39	0.34	0.31	0.27	0.25
SID Lys（%）	1.36	1.23	0.98	0.85	0.73
SID Met+cys（%）	0.74	0.69	0.55	0.55	0.42
SID Thr（%）	0.80	0.73	0.58	0.52	0.46
SID Trp（%）	0.22	0.20	0.17	0.15	0.13

Note 1: Contents per kg of diet for 7-25 kg BW Period: Fe, 150 mg; Cu, 195 mg; Zn, 150 mg; Mn, 30 mg; I, 0.3 mg; Se, 0.3 mg; vitamin A, 12000 IU; vitamin D, 3200 IU; vitamin E, 80 mg; vitamin K3, 32.50 mg; vitamin B1, 2.30 mg; vitamin B2, 6.50 mg; vitamin B6, 5 mg; vitamin B12, 50 μg; nicotinic acid, 45 mg; pantothenic acid, 20 mg, folic acid, 1.50 mg; biotin, 0.15 mg; enzyme preparation and preservatives. Contents per kg of diet for 7-25 kg BW Period: Fe, 120 mg; Cu, 17 mg; Zn, 100 mg; Mn, 25 mg; I, 0.3 mg; Se, 0.2 mg; vitamin A, 5512 IU; vitamin D, 2250 IU; vitamin E, 24 mg; vitamin K3, 3 mg; vitamin B2, 6 mg; vitamin B6, 3 mg; vitamin B12, 24 μg;pantothenic acid, 15 mg, folic acid, 1.20 mg; biotin, 0.15 mg; enzyme preparation and preservatives. Note 2: Nutrient levels were calculated values.

Supplementary Table S9. Information of primers used to perfrom q-PCR

Gene symbol	Forword primer (5'-3')	Reverse primer (5'-3')
COX2	GCACCCCGACATAGAGAGC	CTGCGGAGTGCAGTGTTCT
ND1	CCACTACCAATACCCTACCCTC	TGCGTATTTTGAGTTGGATGCT
ATP6	AAACATCACTAGCCCACTTTCT	TAATGTTGGCTGTCAGTCGTAC
GCG	GAATCAACACCATCGGTCAAAT	CTCCACCCATAGAATGCCCAGT
LncRNA14704	CAAGCCTCATCTGAATCCTCTA	GTCCCACTATCCTCACTGCTAA
LncRNA3569	GAGGAGCAAGATTTAAGAGGCG	CCCTGAGTAGATGATACCCACA
LncRNA6176	ATCTGGTGTTGTCACTGCTTGG	TTAGGAAATATCCATTGTTTGG
Foxj3	GAAACCTCCATACAGTTACGCC	CACTTGTTCAGGGACAGATTAT
MEF2C	GGGTATGGCAATCCCCGAAACT	TTGCTGCCTGGTGGAATAAGAA
SIRT1	TCTTCCCTGAAAGTAAGACCAG	GGCATATTCACCTCCTAACCTA
miR-27b	TTCACAGTGGCTAAGTTCTGC	R-primer (Uni-miR qPCR Primer)
miR-23a	ATCACATTGCCAGGGATTTCC	R-primer (Uni-miR qPCR Primer)
miR-217	TACTGCATCAGGAACTGATTGGAT	R-primer (Uni-miR qPCR Primer)
circRNA290	GATGTGGAATGATTTCTTCTGG	ATCAGATCCAAGTCACGGTCGC
circRNA9210	ACAAACTATTATGGTAATGGAA	CCACAGTTCAACAACAGACCAA
circRNA41	TGGGTTCAATTTCTGACCTTAC	AAACGCATTCACCAGGTATTCA
circRNA118	TCCCGCTCAAAATCACATCAAG	TGCAACTTGCAAACCGCCAGG
circRNA1208	ATATCTATGCAGACACCATCCT	TCTCCTGTGCCCATCATTGTGTG
circRNA154(Divergent)	TACAAATAAATCCCTGCGTAAA	CCTCCTGTCTGGCATAAAGTAA
circRNA154(Convergent)	GTGAGGACGGAACTGATGTGGG	CCTCTGCGGTCGCCACCTTATT
ACTB(Divergent)	ACTCGTCGTACTCCTGCTTGCT	CCCACTGCCTTCTGCTTTCCTC
ACTB(Convergent)	TCTGGCACCACACCTTCT	TGATCTGGGTCATCTTCTCAC
TBP	GATGGACGTTCGGTTTAGG	AGCAGCACAGTACGAGCAA
TOP2B	AACTGGATGATGCTAATGATGCT	TGGAAAAACTCCGTATCTGTCTC

COX2: cytochrome c oxidase subunit 2; *ND1*: NADH dehydrogenase subunit 1; *ATP6:* ATP synthase F0 subunit 6; *GCG*: glucagon; *Foxj3*: forkhead box J3; *MEF2C*: myocyte enhancer factor 2C; *SIRT1*: sirtuin 1; *ACTB*: actin beta; *TBP*: TATA-box binding protein; *TOP2B*: DNA topoisomerase II beta. Uni-miR qPCR Primer included in commercial kits.
